# Does Clear Aligner Treatment Result in Different Patient Perceptions of Treatment Process and Outcomes Compared to Conventional/Traditional Fixed Appliance Treatment: A Literature Review

**DOI:** 10.1055/s-0041-1739441

**Published:** 2021-12-22

**Authors:** Afnan A. Ben Gassem

**Affiliations:** 1 Department of Pediatric Dentistry and Orthodontics, College of Dentistry, Taibah University, Al Madinah Al Munawwarah, Saudi Arabia

**Keywords:** clear aligners, Invisalign, treatment process, treatment outcomes

## Abstract

This study sought to systematically review the literature to determine whether clear aligner treatment results in different patient perceptions of treatment process and outcomes compared with conventional fixed appliance treatment. A systematic review was conducted to identify studies that examined differences in patient perceptions between clear aligners and conventional fixed appliance treatment. Studies were identified through searching relevant terms using PubMed and Embase. Following review of identified articles, key information about the studies including study design, setting, comparison groups, sample size/response rate, study location, primary outcomes, and statistical tests used were extracted. A total of 13 articles were identified that met the inclusion criteria for this study. These studies described a variety of outcomes which were divided into two broad categories: treatment process (pain, chewing, speech, daily routine, etc.) and treatment outcomes (satisfaction level, smile outcome perceptions, etc.). There was the strongest evidence that clear aligners had a positive impact with respect to treatment process compared with fixed orthodontic appliances. This study highlights that clear aligners may be effective for improving treatment-process-related outcomes among orthodontic patients. More studies need to be conducted to determine whether clear aligners have a beneficial impact with respect to treatment outcomes.

## Introduction

In 1946 Kesling first introduced the concept of clear removable orthodontic appliances to move misaligned teeth. 1 Initially, minor cases of crowding or spacing were treated with clear aligners, but with the development of aligner materials and enhancement of computer design software for tooth movement, the indication of clear aligners has been greatly enlarged and proved to be successful in treating a variety of mild to severe malocclusions. 2 3 As opposed to traditional fixed orthodontic appliances (e.g., MBTTM bracket systems and Damon bracket systems), clear aligners are transparent and removable, and hence patients may prefer the usage of clear aligners for aesthetic reasons. The usage of clear aligners as an orthodontic treatment option became most common in 1980 following the introduction of Invisalign. 4 5

While clear aligners may be appealing to patients, it is difficult to be certain about the effectiveness of these devices compared with traditional orthodontic devices. A systematic review suggests that clear aligners may be effective for certain types of orthodontic malocclusions such as aligning and leveling arches, and controlling anterior intrusion, posterior buccolingual inclination, and upper molar bodily movements, but not effective for other issues such as anterior buccolingual inclination and correction of rotated teeth. 6 A meta-analysis found that the only improvement observed among clear aligners compared with traditional treatment was shortened treatment duration and chair time. 7 However, there is evidence that clear aligners may result in improved periodontal outcomes compared with traditional orthodontic treatment. 8

While most of the studies discussed above deal with the objective outcomes of clear aligner treatment, it may also be important to consider the subjective experience of patients using clear aligner treatment compared with traditional fixed orthodontic appliances. It is important to make comparisons between these different types of devices because different patient perceptions may impact clinical recommendations for the type of appliance that should be used. There are other factors that may impact the preference of a patient for different types of orthodontic treatments such as the aesthetic impact of the device as well as the pain caused by the device. Clear aligners may be seen as more aesthetically pleasing than other devices. In a study that sought to assess preferences for different orthodontic appliances among orthodontic patients, no benefits were observed for certain outcomes including sleeping, absences from work or school, difficulties in daily psychosocial improvement, social performance, and concentration during work or studies. 8 There is some evidence that there may be different perceptions of individuals depending on the type of appliance that they are wearing. For example, in a study it was found that those who wore fixed gold appliances or clear aligners were perceived to have more intellectual ability than those who wore ceramic or steel fixed appliances. 9

Therefore, subjective patient outcomes may be an important element of the experience of treatment with the clear aligner technology, which should be investigated. A previous systematic review examined the impact of clear aligners on oral health-related quality of life (OHRQoL). This review only found two studies that met the inclusion criteria of the study. The authors were only able to derive weak evidence from this review suggesting that clear aligners might cause less disturbance while eating compared with conventional appliances. 10 Other systematic reviews reported reduced speech difficulties and pain especially in the early stages of treatment. 11 12 13

Previous literature reviews focused on the impacts of clear aligners on either objective treatment outcomes or very specific subjective outcomes (pain, oral-health quality of life). A comprehensive review of subjective outcomes across the timespan of using clear aligners may provide a more complete picture of what patients' experiences are with respect to the use of clear aligners. This information can be useful clinically for providing patients with the most comprehensive information for the treatment. Because of the need to understand the subjective experience of patients depending on the type of orthodontic device they received for their treatment, this study sought to systematically review the literature to answer the research question of whether clear aligner treatment results in different patient perceptions of treatment process and outcomes compared with conventional fixed appliance therapy. The review builds on previous work by considering all subjective outcomes and dividing these outcomes into those related to treatment process and outcomes following treatment.

## Methods

We followed the Preferred Reporting Items for Systematic Reviews and Meta-Analyses (PRISMA) guidelines for reporting findings from systematic reviews for this study. 14

### Inclusion/Exclusion Criteria

This literature review sought to summarize studies that dealt with patients' subjective experience of using clear aligners compared with other orthodontic devices (e.g., labial/buccal fixed and lingual fixed orthodontic appliances). Specific inclusion criteria for this literature review were: (1) publication in English language, (2) individual level data (meaning studies that collected data from individuals rather than using aggregate data), (3) clinical trials or observational study design (including both prospective and retrospective studies), (4) subjective patient outcomes including patient satisfaction or different psychometric tools, and (5) human studies. Specific exclusion criteria included: (1) studies lacking a control group using an orthodontic device other than clear aligners, (2) *in vitro* and animal studies, (3) case reports/case series, and (4) editorials, opinions, narrative reviews, and technique description articles, without reported sample. Table 1 shows the PICO (population, intervention, control, and outcomes) table for this study.

**Table 1:** PICO table for literature review

Population	This review examined human studies including pediatric and adult population.
Intervention	Clear aligners
Comparator	Traditional fixed appliances
Outcome	Treatment process (pain, chewing, speech, daily routine, etc.) and treatment outcomes (satisfaction level, smile outcome perceptions, etc.).

Abbreviation: PICO, population, intervention, control, and outcomes.

### Search Strategy

We reviewed studies that were published between January 1, 2000, and July 31, 2020. Initial searches were performed using PubMed and Embase. Of these databases, we searched for the following terms:

1. Clear aligner treatment.
2. Invisalign treatment.
3. Clear aligner patient satisfaction.
4. Invisalign patient satisfaction.
5. Clear aligner malocclusion.
6. Invisalign patient malocclusion.
7. Clear aligner outcome.
8. Invisalign outcome.
9. Clear aligner process.
10. Invisalign process.
11. Clear aligner oral health quality of life.
12. Invisalign patient oral health quality of life.

Mesh terms included: appliance, removable orthodontic; appliances, removable orthodontic; orthodontic appliance, removable; removable orthodontic appliance; removable orthodontic appliances; clear aligner appliances; aligner appliance, clear; aligner appliances, clear; appliance, clear aligner; appliances, clear aligner; clear aligner appliance; clear dental braces; brace, clear dental; braces, clear dental; clear dental brace; dental brace, clear; dental braces, clear; Invisalign.

Following these searches, potentially relevant titles were identified. The abstracts from the selected titles were reviewed to confirm their eligibility and retrieve the full texts. Finally, the full texts from these articles were reviewed to conclude whether the studies met the inclusion and exclusion criteria for the study. Two reviewers reviewed the identified titles, abstracts, and full texts. The two reviewers initially agreed about inclusion versus exclusion for over 90% of the identified articles. Disagreements about whether the articles met this study's inclusion criteria were dealt with through meetings among the reviewers.

Relevant literature reviews and meta-analyses identified during the searches were reviewed to find articles that potentially met the inclusion criteria for this study. Additionally, searches were performed using Google Scholar to find articles from the gray literature to be included. While searching Google Scholar the same search terms described above were used.

After articles that met the studies' inclusion criteria were identified, a database that contained information extracted from these articles was created. The information that was extracted included (when available) articles' authors, study design, setting, comparison groups, sample size/response rate, study location, the primary outcomes that were measured, statistical tests used, the study's inclusion and exclusion criteria, and the years the study was conducted. Finally, information regarding the study's major outcomes was collected. The Health Canada Quality Assessment (HCQA) tool for observational and experimental studies was used to assess the overall quality of included studies. The tool for observational studies was used for all except one study, for which the experimental tool was used. 14 The HCQA tool was used because it allows for the quality of both observational and experimental studies to be assessed in a systematic manner. Quality of evidence in the included studies was assessed using the GRADE. 15

## Results

### Included Studies

**Fig. 1:**
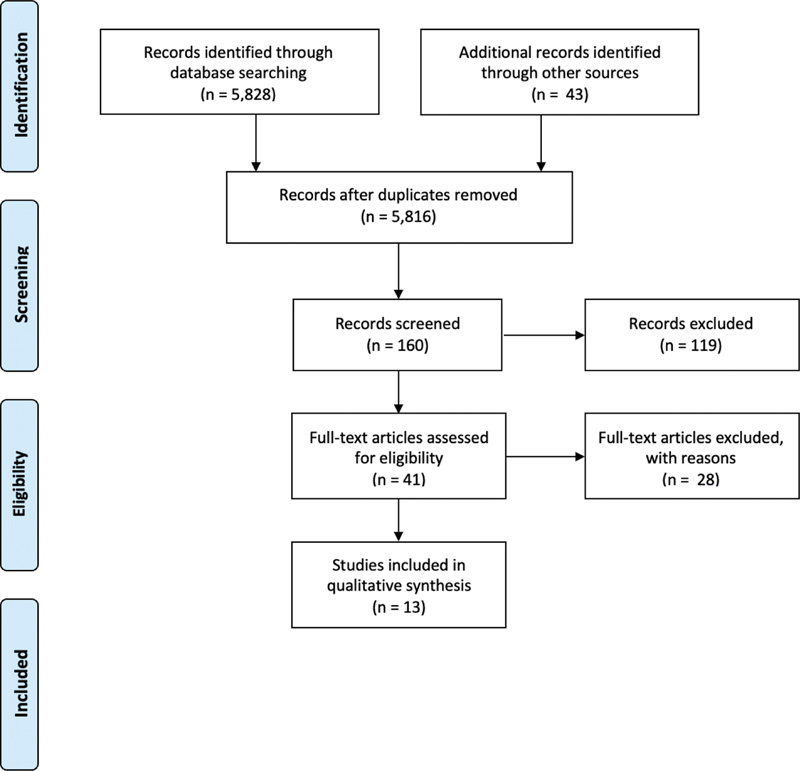
Flow diagram of article review.

As shown in Fig. 1 , a total of 5,816 article titles were reviewed for inclusion in the literature. Based on these titles, 160 potentially relevant abstracts were reviewed. Of these, 39 articles had their full texts retrieved and reviewed of which six were identified that met the inclusion criteria for the current study. An additional six articles were added from a review of the gray literature about the topic. Finally, one additional article was added that was found from reviewing relevant literature reviews. This resulted in an overall 13 articles that were included for the review. Articles that were excluded following full-text review are shown in Supplementary Table S1 (available only in the online version).

### Study Characteristics

As shown in Table 2 , most studies used an observational study design. Only one study was a randomized trial. 16 Of the observational studies, a total of two were retrospective, 17 18 seven were prospective or longitudinal, 19 20 21 22 23 24 25 and three were cross-sectional. 24 25 26 All studies compared Invisalign or other clear aligner technology to traditional, fixed orthodontic appliances. Some studies compared specific brands of products. 23 24 25 Sample sizes varied widely from a low of 25 27 to a high of 145. 28 Many studies did not report the response rate or how many of those recruited completed the study. Among the studies that did, rates tended to be fairly high, generally greater than 80%. 17 28

**Table 2:** Characteristics of studies

Author(s)	Study design	Setting	Comparison group	Sample size/response rate	Study location	Primary outcome(s)	Statistical tests used	Quality of the evidence (GRADE)
Alajmi et al, 2020	Observational retrospective study	Not stated	Invisalign patients compared with conventional buccal fixed appliance patients	60	Kuwait	Treatment process: speech difficulty, chewing ability, daily routine. Treatment outcome: overall treatment satisfaction	Chi-square test, Fisher's exact test, and the Z test	Moderate
Miller et al, 2007	Prospective, longitudinal cohort study	Private orthodontic offices	Invisalign aligners compared with preadjusted fixed appliances	60	United States	Treatment process: functional, psychosocial, and pain-related outcomes	Fisher exact tests, Wilcoxon rank sum tests, and two-sample *t-* test	Moderate
Azaripour et al, 2015	Cross-sectional study	Not stated	Invisalign compared with fixed orthodontic appliances	100 (out of 139 patients screened)	Not stated	Treatment outcome: patient satisfaction	Linear mixed models, Mann– Whitney U-test, and Fisher's exact test	Low
Gao et al, 2021	Prospective study	Hospital	Clear aligners and fixed appliances	110	Gago	Treatment process: pain perception, anxiety, oral health-related quality of life	Two-way analysis of variance	Moderate
Antonio-Zancajo et al, 2020	Prospective study	Not stated	Conventional brackets, conventional low-friction brackets, lingual brackets, and clear aligners	120	Not stated	Treatment process: pain and oral-health-related quality of life	Analysis of variance	Moderate
Flores-Mir et al, 2018	Observational cross-sectional study	Practice practices and a university clinic	Patients treated with brackets or Invisalign	145, 84.1%	Canada	Treatment process: quality of life, chewing Treatment outcome: patient satisfaction	Multivariate analysis of variance	Low
Christou et al, 2020	Retrospective case–control	University orthodontic clinic	Invisalign clear aligners and traditional fixed appliances	59	United States	Treatment outcome: smile scores	Mann–Whitney U-test and the Wilcoxon *t* -test	Moderate
White, 2015	Randomized prospective trial	Graduate orthodontic clinic	Invisalign compared with traditional fixed appliances	41 out of 240 patients screened for inclusion	United States	Treatment process: pain levels and sleep disturbances	Nonparametric Mann–Whitney test	High
Carrol, 2007	Cross-sectional study	University dental center and private orthodontic clinic	Invisalign and fixed orthodontic appliances	25	United States	Treatment process: pain and impacts on daily life	Fisher exact and Wilcoxon rank sum tests	Very low
Nicholson, 2011	Cohort study	Private orthodontic practices	Invisalign and fixed appliances	63 out 74 patients who filled out some of survey	United States	Treatment process: quality of life	Pearson's chi-squared test or Fisher's exact test	Moderate
Rucker, 2012	Prospective, longitudinal cohort study	Not stated	Invisalign and fixed Damon appliances	60	United States	Treatment process: patient discomfort	Not stated	Moderate
Lawton, 2003	Prospective, longitudinal study	University orthodontic clinics	Invisalign compared with edgewise appliances	129 from 137 initial recruited	United States	Treatment process: psychosocial impacts of treatment	Wilcoxon rank sum and two-sample *t* -tests	Moderate
Shalish et al, 2012	Prospective study	University orthodontic clinic and private orthodontic clinic	Buccal, lingual, and Invisalign devices	68 patients	Palestine/Israel	Treatment process: patients' perception of pain, oral dysfunction, eating disturbances, general activity parameters, and oral symptoms	Two-way analysis of variance	Moderate

The studies were completed in a wide variety of settings including six in university health centers, five in private orthodontic clinics, and one in a hospital. Some studies were conducted in multiple settings. Four studies did not report their setting. Seven of the studies were conducted in the United States and two studies did not state where they were conducted. The remaining four studies were conducted in different countries.

Table 2 also shows that a wide variety of outcomes were compared between the different studies. Broadly, we divided outcomes into those related to treatment process (pain, chewing, speech, daily routine, etc.) and those related to treatment outcomes (satisfaction level, smile outcome perceptions, etc.). Individual studies could have both categories of outcomes. Eleven of the studies had treatment process outcomes as their primary outcome and three studies had treatment outcomes as their primary outcome. Several different statistical techniques were used to analyze the outcomes from the studies. Chi-square analysis was frequently used to compare the distribution of categorical outcomes between the exposure categories and *t* -tests or analysis of variance tests were frequently used to compare mean scores for continuous outcomes.

As shown in Table 4 , using the GRADE criteria, 9 of the 13 studies were ranked as being moderate meaning that it was judged that the effect measure in the study is likely close to the actual effect in the real world. Two studies were ranked as low quality, one was ranked as very low quality, and one study was ranked as high quality (additional characteristics of the included studies are provided in Supplementary Table S2 [available only in the online version]).

### Treatment Process

Table 3 displays the results of the selected studies that are related to the treatment process and outcome of orthodontic treatment. There were consistent findings with respect to chewing with two studies finding less chewing difficulty in the clear aligner group. 17 28 These consistent findings were noticed despite the fact that there were differences between these studies. Alajmi et al 17 matched participants according to specific characteristics, while Flores-Mir et al 28 just utilized a convenience sample. Alajmi et al's 17 design may have been better able to control for potential confounding factors. However, Flores-Mir et al 28 had a larger sample size, which may have resulted in a more precise assessment of the effect of clear aligners on chewing. Alajmi et al 17 found that those who used clear aligners had more difficulty speaking and that they experienced no change in their daily routine.

**Table 3:** Quality of studies

Reference									
Observational studies									
	Were the inclusion and exclusion criteria for study participation reported (e.g., age greater than 50 years, no history of heart disease)?	Was attrition numerically reported?	Were the reasons for withdrawals and dropouts provided?	Was the methodology used to measure the exposure reported?	Were sample size justification, power description, or variance and effect estimates provided?	Was the exposure assessed more than once?	Was the methodology used to measure the health outcome reported?	Was the health outcome verified (e.g., through assessment of medical records, confirmation by a health practitioner)?	Were the outcome assessors blinded to the exposure status?
Alajmi et al, 2020	1	1	1	1	0	0	1	0	0
Miller et al, 2007	0	1	1	1	0	0	1	0	0
Azaripour et al, 2015	1	1	1	1	0	0	1	1	0
Gao et al, 2021	1	1	1	1	1	0	1	0	0
Antonio-Zancajo et al, 2020	1	1	1	1	1	0	1	0	0
Flores-Mir et al, 2018	1	1	1	1	0	0	1	0	0
Carrol, 2007	1	1	1	1	1	0	1	0	0
Nicholson, 2011	1	1	1	1	0	0	1	0	0
Rucker, 2012	1	0	1	1	0	0	1	0	0
Lawton, 2003	1	1	0	1	0	0	1	0	0
Shalish et al, 2012	1	0	0	1	0	0	1	0	0
Christou et al, 2020	1	1	1	1	1	0	1	1	0
Experimental study									
	Were the inclusion and exclusion criteria for study participation reported (e.g., age greater than 50 years, no history of heart disease)?	Was the study described as randomized?	Was the randomization method reported?	Was the randomization appropriate?	Was the allocation concealed?	Were the study subjects blinded to the intervention received?	Were the researcher personnel blinded to the intervention received by the subjects?	Was attrition numerically reported?	Were the reasons for withdrawals and dropouts provided?
White, 2015	1	1	0	0	0	0	0	1	1

**Table:** 

Reference							
Observational studies							
Were the subjects in different exposure groups compared at baseline?	Was the statistical significance of the trend reported?	Were key confounders related to subjects' demographics accounted for in the statistical analysis?				Total score	Quality
1	1	0				7	Higher
1	1	0				6	Lower
1	1	1				9	Higher
0	1	1				8	Higher
1	1	0				8	Higher
1	1	1				8	Higher
0	1	0				7	Higher
0	1	1				7	Higher
0	1	0				5	Lower
0	1	0				5	Lower
1	1	0				5	Lower
1	1	0				9	Higher
Experimental study							
Was the type of exposure described?	Was the amount of exposure described?	Was the methodology used to measure the health effect reported?	Was between-group statistical analysis of the health effect reported?	Was an intention-to-treat analysis conducted?	Were potential confounders of the food health relationship considered?		
1	1	1	1	0	1	9	Higher

Note: 1 = yes; 0 = no.

Five of the six studies that assessed pain-related outcomes found higher pain in the conventional device groups 16 17 19 20 25 , and one did not. 27 Five of these studies used a validated tool to measure pain. 19 20 25 27 28 , White 16 used a daily diary to assess pain and it was not clear whether this method was validated. Carrol's 27 study had a smaller sample size than the other studies that examined the relationship between the use of clear aligners and pain, which may have meant that the study was underpowered to find the differences identified in the other studies.

Of the four studies that assessed quality of life, three found that the clear aligner technology had a positive impact 17 19 20 and one study found no difference between the two groups. 20 All four of the studies used a validated OHRQoL questionnaire. In Miller et al 16 improvement was observed both for the impact scores and psychosocial elements of the OHRQoL score. In Antonio-Zancajo et al, 29 the greatest beneficial impacts of clear aligners were observed with respect to physical pain, psychological discomfort, and physical/psychological/social disability elements of the score. Carrol 27 did not present the individual elements of the quality-of-life score.

White 16 found no significant difference with respect to sleep quality between the two groups. Rucker 23 found more discomfort among the traditional appliance group. While Lawton 24 compiled questions from previously validated questionnaires to assess psychosocial impacts and found few differences with respect to the psychosocial effects of treatment between the groups. Gao et al 20 found lower anxiety among those using clear aligners using a validated method.

### Treatment Outcomes

Of the three studies that assessed patient satisfaction, one found more satisfaction among the clear aligner group 26 and two studies found no difference. 17 28 All three of these studies used validated questionnaires for data collection. It is not clear what factors may have accounted for the variation in findings between these three studies. With respect to the negative findings from the Alajmi et al 17 study, these findings may have been due to the shorter period of follow-up compared with Azaripour et al. 26 It should also be noted that in Azaripour et al 26 the mean age of the conventional fixed appliance group was significantly lower than the clear aligner group, which may have contributed to the differing findings due to confounding by age. Christou et al 18 found better smile scores in the traditional appliance group compared with the clear aligner group. It was not clear whether the method used for assessment of smile score by Christou et al 18 was validated.

### Quality Assessment

We assessed the quality of the studies included in this review using the HCQA tool. These scores are shown in Table 4 . Nine of the 13 studies were assessed as being higher quality; however, most of the scores were close to the cut-off between higher and lower quality. Some of the reasons for the lower scores are understandable given the nature of these studies. For example, all studies were graded as “no” for whether the exposure was assessed more than once. Given the nature of the exposure (the type of dental appliance), multiple assessments were not likely necessary or possible. Other consistent score elements that contributed to lower scores included the lack of clinical evaluation—most outcomes were assessed with self-reports only—and the lack of controlling for confounding. Most studies did collect data about confounders and compared them at baseline, but did not control for these confounders in the analysis, likely due to the small sample sizes, which limits the ability to control for confounding. Also, most studies used basic statistical methods and did not use regression, which can allow for the consideration of potential confounders. Additionally, many studies did not include justifications of their sample sizes, which means that some studies may have been underpowered. As noted above, underpowered studies may explain some variation in findings between studies.

**Table 4:** Outcomes of studies

Author(s)	Major findings
Alajmi et al, 2020	More difficulty with speech and less restriction on chewing ability among the clear aligner group. No differences with respect to daily routine, use of analgesics, and overall treatment satisfaction.
Miller et al, 2007	Patient in the Invisalign group reported fewer negative impacts of treatment in terms of quality of life and less pain during the first week of treatment.
Azaripour et al, 2015	More patient satisfaction among patient treated with Invisalign compared with fixed orthodontic appliances.
Gao et al, 2021	Lower pain and anxiety levels among those receiving clear aligners. Oral health quality-of-life scores were higher in the fixed appliance group.
Antonio-Zancajo et al, 2020	Lingual orthodontic patients had lower levels of pain and less impact on their oral quality of life. No major differences seen for the clear aligner group.
Flores-Mir et al, 2018	More satisfaction with respect to eating and chewing in the Invisalign group. No overall difference in patient satisfaction between the two groups.
Christou et al, 2020	In general, better smile outcomes were observed among the fixed appliance group compared with the Invisalign group.
White, 2015	Higher pain levels reported in convention fixed appliance group compared with the Invisalign group. No significant differences in sleep disturbances.
Carrol, 2007	No significant differences in pain and impacts on daily life between the Invisalign and fixed orthodontic appliances groups.
Nicholson, 2011	Fewer negative life impacts among the Invisalign grouped compared with the fixed appliances group. No differences in quality of life.
Rucker, 2012	More discomfort among the fixed Damon appliances group compared with the Invisalign group.
Lawton, 2003	Few differences in terms of psychosocial impacts between the Invisalign compared with edgewise appliances groups.
Shalish et al, 2012	More pain and oral dysfunction among those with lingual appliances. Full recovery was not reached among more lingual and buccal patients.

### Bias Assessment

As shown in Table 5 , all studies were ranked as being at either a moderate or a serious risk of bias. Confounding was one of the main concerns in many of the studies that resulted in several of them being ranked as having a serious risk of bias. This concern about confounding is due to the fact that most of the studies were not randomized and the studies often did not use multivariate methods to control for confounding. Many studies also did not examine whether there were differences between the clear aligner and comparison groups. All studies were ranked as being at a moderate risk of bias due to selection, because of the fact that convenience samples were frequently used. Bias due to classification and maintaining the treatment was not a concern because of the nature of the intervention.

**Table 5:** ROBINS-I bias assessment for included studies

Paper	Bias due to confounding	Bias in selection of participants into the study	Bias in classification of interventions	Bias due to deviations from intended intervention	Bias due to missing data	Bias in measurement of outcomes	Bias in selection of the reported result	Overall
Alajmi et al, 2020	Serious	Moderate	Low	Low	Low	Moderate	Low	Serious
Miller et al, 2007	Serious	Moderate	Low	Low	Low	Moderate	Low	Serious
Azaripour et al, 2015	Low	Moderate	Low	Low	Low	Moderate	Low	Moderate
Gao et al, 2021	Low	Moderate	Low	Low	Low	Moderate	Low	Moderate
Antonio-Zancajo et al, 2020	Serious	Moderate	Low	Low	Low	Moderate	Low	Serious
Flores-Mir et al, 2018	Low	Moderate	Low	Low	Low	Moderate	Low	Moderate
White, 2015	Low	Moderate	Low	Low	Low	Moderate	Low	Moderate
Carrol, 2007	Moderate	Low	Low	Low	Low	Moderate	Low	Moderate
Nicholson, 2011	Low	Low	Low	Low	Low	Moderate	Low	Moderate
Rucker, 2012	High	Moderate	Low	Low	Low	Moderate	Low	Serious
Lawton, 2003	Serious	Moderate	Low	Lowe	Low	Moderate	Low	Serious
Shalish et al, 2012	Serious	Moderate	Low	Low	Low	Moderate	Low	Serious

## Discussion

### Summary of Evidence

This literature review examined the differences in the subjective perceptions of treatment process and outcomes among patients with clear aligners compared with traditional orthodontic devices. The subjective outcomes were divided into those impacting the treatment process and those impacting the treatment outcome. In general, it was found that more studies support the fact that clear aligners have a positive impact with respect to pain 16 17 19 20 25 27 and quality of life. 17 20 22 28 With respect to quality-of-life scores, different elements of these were found to be improved for those using clear aligners including psychosocial, physical pain, 24 psychological discomfort, 23 and physical/psychological/social disability. 19 With respect to sleep discomfort and anxiety, there were too few studies to draw firm conclusions about the impact of clear aligners. 16 20 With respect to chewing, there were two studies that found a positive effect of clear aligners. 17 28

Overall there were fewer studies that examined the role of clear aligners compared with conventional fixed appliances with respect to treatment outcomes, so it is not possible to completely assess the impact of clear aligners on these outcomes like patient satisfaction and smile scores. 18 26 28 However, more of the studies dealing with patient satisfaction did not find a beneficial effect of clear aligners. The one study dealing with smile score also found a beneficial impact. 18 Ultimately, it appears to be the case that there are process-related beneficial effects of clear aligners for some variables such as elements of quality of life including pain, but the evidence with respect to treatment outcomes is less certain. Some of this lack of evidence may be due to the fact that there are fewer studies that examined clinical outcomes compared with process outcomes.

These findings are potentially useful for clinical practice. There are many reasons why a patient may decide to use a specific orthodontic appliance over others. In particular, they may be curious about both the experience of the appliance and the ultimate outcome of using the appliance. Although many of the studies suggested that clear aligners are more useful than traditional fixed orthodontic appliances for improving the treatment experience, but not necessarily better for improving clinical outcomes, there is not enough evidence to make firm conclusions about this effect because of the limited number of studies. Health care providers can make different recommendations based on the findings from this literature review. Patients may also want to consider these findings as they decide which appliance is best suited for them.

### Risk of Bias

The risk of bias in these studies should be considered. In all studies, the study population consisted of convenience samples collected from populations served by different clinics, practices, and hospitals. Additionally, in general the studies had fairly small sample sizes, therefore inadequate sample may be a concern in many of these studies. Five 16 18 20 27 29 , of the 13 studies included found that their sample sizes were not adequate.

With respect to study design, the role of reverse causality is not as high because many of the studies were longitudinal. The generalizability of these findings should also be considered. Most studies included a fairly even distribution of males and females. Most studies focused on older populations with mean ages in the 20s and 30s, with two exceptions. 16 24 Therefore, the findings from this review should not be necessarily generalized to younger populations.

### Limitations

With respect to limitations, consideration should be given both to the issues with the studies included in this review and also to the review itself. With respect to the studies, as noted above, many of these studies are vulnerable to bias for three primary reasons: (1) small sample sizes, (2) nonexperimental study designs, and (3) limited controls for confounding. Taken together, these factors may result in some of the studies' findings not being generalizable to larger populations and lack of confidence both in the findings of associations and in the findings of no association. While the quality scores for many of the included studies were generally high, these problems were persistent for many of the studies.

Additionally, because this study focused on subjective outcomes, the actual methods for measuring these outcomes can differ between studies. Even when multiple studies were examining one conceptual outcome, they sometimes used different operational definitions of this outcome.

With respect to this review itself, there are also some limitations. The focus was on a wide variety of different outcomes, and hence for most specific outcomes, there were relatively few studies that actually examined that particular outcome. This limited number of studies for most outcomes prevented making firm conclusions for many. Additionally, studies conducted before the year 2000 were not considered for review and hence some studies that could have potentially been included in this study were not captured; however, it was felt that older research would have been less relevant especially with the rapid advancement of clear aligner therapy and orthodontic treatment in general. Publication bias also remains a concern, as researchers may be less likely to publish studies that do not have positive or negative findings. Another limitation of this review was the fact that it was not preregistered. Additionally, although the databases searched yielded several relevant articles, it is possible that additional database searching may yield more results. Finally, because of the heterogeneity of the outcomes measured, it was not possible to do a formal meta-analysis.

## Conclusion

These findings highlight that clear aligners may improve the patients' perceptions of the treatment process and outcomes. However, there is not enough evidence to make a final conclusion about treatment outcomes. Additionally, other factors should be considered when making recommendations about the best orthodontic course of treatment such as the type of tooth movements required to achieve the best clinical results which may outweigh any perceived subjective preference for such a treatment modality. There is a need for more studies exploring subjective outcomes among clear aligners compared with traditional devices, especially studies related to difficulty speaking, chewing, sleeping, and discomfort. These future studies should focus on recruiting more patients, utilizing experimental study designs, and recruiting generalizable populations, not just convenience samples. Conducting such studies will allow for making firmer conclusions about the treatment and outcome effects of clear aligners compared with traditional fixed appliances.
