# Supplementary material for: Does Clear Aligner Treatment Result in Different Patient Perceptions of Treatment Process and Outcomes Compared to Conventional/Traditional Fixed Appliance Treatment: A Literature Review
Source: Eur J Dent. 2021 Dec 22;16(2):274–85. doi: 10.1055/s-0041-1739441 (PMC9339930; doi:10.1055/s-0041-1739441)
Supplement: Supplementary file 1 — Supplementary Material [file 10-1055-s-0041-1739441-s2161652.pdf]

Supplementary Table S1 Excluded articles

Citation
Martorelli M, Gerbino S, Giudice M, Ausiello P. A comparison between customized clear and removable orthodontic appliances manufactured using RP and CNC techniques. Dent Mater J 2013;29(2):e1–e10
Hennessy J, Garvey T, Al-Awadhi EA. A randomized clinical trial comparing mandibular incisor proclination produced by fixed labial appliances and clear aligners. Angle Orthod 2016;86(5):706–712
Wiedel AP, Bondemark L. A randomized controlled trial of self-perceived pain, discomfort, and impairment of jaw function in children undergoing orthodontic treatment with fixed or removable appliances. Angle Orthod 2016;86(2):324–330
Charalampakis O, Iliadi A, Ueno H, Oliver DR, Kim, KB. Accuracy of clear aligners: a retrospective study of patients who needed refinement. Am J Orthod Dentofac Orthop 2018;154(1):47–54
Sollenius O, Petrén S, Bondemark L. An RCT on clinical effectiveness and cost analysis of correction of unilateral posterior crossbite with functional shift in specialist and general dentistry. Eur J Orthod 2020;42(1):44–51
Forde K, Storey M, Littlewood SJ, Scott P, Luther F, Kang J. Bonded versus vacuum-formed retainers: a randomized controlled trial. Part 1: stability, retainer survival, and patient satisfaction outcomes after 12 months. Eur J Orthod 2018;40(4):387–398
Dai FF, Xu TM, Shu G. Comparison of achieved and predicted tooth movement of maxillary first molars and central incisors: first premolar extraction treatment with Invisalign. Angle Orthod 2019;89(5):679–687
Sharma K, Mangat S, Kichorchandra MS, Handa A, Bindhumadhav S, Meena M. Correlation of orthodontic treatment by fixed or myofunctional appliances and periodontitis: a retrospective study. J Contemp Dent Pract 2017;18(4):322–325
Park, J. H., & Kim, T. W. Deep-bite correction using a clear aligner and intramaxillary elastics. J Clin Orthod 2009;43(3):152–157
Grünheid T, Gaalaas S, Hamdan H, Larson BE. Effect of clear aligner therapy on the buccolingual inclination of mandibular canines and the intercanine distance. Angle Orthod 2016;86(1):10–16
Al-Moghrabi D, Johal A, O'Rourke N, Donos N, Pandis N, Gonzales-Marin C, Fleming PS. Effects of fixed vs removable orthodontic retainers on stability and periodontal health: 4-year follow-up of a randomized controlled trial. Am J Orthod Dentofac Orthop 2018;154(2):167–174
Dasy H, Dasy A, Asatrian G, Rózsa N, Lee HF, Kwak JH (2015). Effects of variable attachment shapes and aligner material on aligner retention. Angle Orthod 2015;85(6):934–940
Gu J, Tang JS, Skulski B, Fields Jr HW, Beck FM, Firestone AR, Deguchi T. Evaluation of Invisalign treatment effectiveness and efficiency compared with conventional fixed appliances using the Peer Assessment Rating index. Am J Orthod Dentofac Orthop 2017;151(2):259–266
Eissa O, Carlyle T, El-Bialy T. Evaluation of root length following treatment with clear aligners and two different fixed orthodontic appliances. A pilot study. J Orthod Sci 2018;7:11
Wiedel AP, Bondemark L. Fixed versus removable orthodontic appliances to correct anterior crossbite in the mixed dentition—a randomized controlled trial. Eur J Orthod 2015;37(2):123–127
Kravitz ND, Kusnoto B, BeGole E, Obrez A, Agran B. (2009). How well does Invisalign work? A prospective clinical study evaluating the efficacy of tooth movement with Invisalign. Am J Orthod Dentofac Orthop 2009;135(1):27–35
Caruso S, Nota A, Ehsani S, Maddalone E, Ojima K, Tecco S. Impact of molar teeth distalization with clear aligners on occlusal vertical dimension: a retrospective study. BMC Oral Health 2019;19(1):1–5
Paes da Silva S, Pitchika V, Baumert U, Wehrbein H, Schwestka-Polly R, Drescher D, Wichelhaus A. Oral health-related quality of life in orthodontics: a cross-sectional multicentre study on patients in orthodontic treatment. Eur J Orthod 2020;42(3):270–280
Djeu G, Shelton C, Maganzini A. Outcome assessment of Invisalign and traditional orthodontic treatment compared with the American Board of Orthodontics objective grading system. Am J Orthod Dentofac Orthop 2005;128(3):292–298
Papakostopoulou M, Migliorati M, Calzolari C, Gallo F, Drago S. Patients' expectations assessment in orthodontic treatment: findings from a questionnaire survey. Minerva Stomatol 2016;65(6):343–352
Jäderberg S, Feldmann I, Engström C. Removable thermoplastic appliances as orthodontic retainers—a prospective study of different wear regimens. Eur J Orthod 2012;34(4):475–479
Lanteri V, Farronato G, Lanteri C, Caravita R, Cossellu G. The efficacy of orthodontic treatments for anterior crowding with Invisalign compared with fixed appliances using the Peer Assessment Rating Index. Quintessence Int 2018;49(7):581–587
Carter LA, Geldenhuys M, Moynihan PJ, Slater DR, Exley CE, Rolland SL. The impact of orthodontic appliances on eating—young people's views and experiences. J Orthod 2015;42(2):114–122
Miethke RR, Wronski C. What can be achieved with removable orthodontic appliances? J Orofac Orthop 2009;70(3):185–199
Alajmi S, Shaban A, Al-Azemi R. Comparison of short-term oral impacts experienced by patients treated with Invisalign or conventional fixed orthodontic appliances. Med Princ Pract 2020;29(4):382–388

(*Continued*)

 © 2021. The Author(s).

Supplementary Table S1 (*Continued*)

Citation
Livas C, Delli K, Pandis N. "My Invisalign experience": content, metrics and comment sentiment analysis of the most popular patient testimonials on YouTube. Prog Orthod 2018;19(1):1–8
Kankam H, Madari S, Sawh-Martinez R, Bruckman KC, Steinbacher DM. Comparing outcomes in orthognathic surgery using clear aligners versus conventional fixed appliances. J Craniofac Surg 2019;30(5):1488–1491
Schaefer I, Braumann B. Halitosis, oral health and quality of life during treatment with Invisalign® and the effect of a low-dose chlorhexidine solution. J Orofac Orthop 2010; 71(6):430–441
Brignardello-Petersen R. Moderate-to-high levels of satisfaction and no important concerns in patients who received treatment with clear orthodontic aligners. J Am Dent Assoc 2019;150(2):e20
Nedwed V, Miethke RR. (2005). Motivation, acceptance and problems of Invisalign® patients. J Orofac Orthop 2005;66 (2):162–173
Walton DK, Fields HW, Johnston WM, Rosenstiel SF, Firestone AR, Christensen JC. Orthodontic appliance preferences of children and adolescents. Am J Orthod Dentofac Orthop 2010;138(6):698-e1.
Pacheco-Pereira C, Brandelli J, Flores-Mir C. Patient satisfaction and quality of life changes after Invisalign treatment. Am J Orthod Dentofac Orthop 2018;153(6):834–841
Pogal-Sussman-Gandia CB, Tabbaa S, Al-Jewair T. Effects of Invisalign® treatment on speech articulation. Int Orthod 2019;17 (3):513–518

Supplementary Table S2 Additional characteristics of included studies

Author(s)	Inclusion criteria	Exclusion criteria	Year
Alajmi et al, 2020	Treatment involved both jaws<br>Treatment did not involve tooth extraction<br>Crowding or spacing limited to 1–4 mm<br>Angle class I molar relationship without skeletal discrepancy<br>Absence of vertical or transverse discrepancy<br>Absence of periodontal disease<br>Full complement of dentition except for third molars	Not stated	Not stated
Miller et al, 2007	At least 18 years of age<br>In good general health<br>Received treatment in both dental arches	Not stated	Not stated
Azaripour et al, 2015	FOA or Invisalign for at least 6 months<br>Modified sulcus bleeding index (SBI) $\leq$20% prior to orthodontic treatment<br>Approximal plaque index (API) $\leq$25% prior to orthodontic treatment<br>Declaration of consent	History of periodontitis<br>Diseases that affect periodontal health<br>Smoking<br>Pregnancy<br>Withdrawal of consent<br>Participation in another clinical trial	Not stated
Gao et al, 2021	Ages greater than 18 years old<br>Good general health<br>Receiving orthodontic treatment in both arches	Untreated dental caries<br>Periodontal diseases<br>Missing teeth in need of prosthetic rehabilitation<br>Previous orthodontic treatment<br>Receiving lingual orthodontic treatment	Not stated
Antonio-Zancajo et al, 2020	Patients between 18 and 40 years of age<br>With permanent dentition<br>Without previous orthodontic treatment<br>No previous extractions except third molars<br>Dental bone discrepancy between –2 and –6 mm in both arches	Patients with deciduous teeth or in the process of dental replacement<br>Patients in need of orthodontic surgical treatment or dental extractions due to treatment<br>Patients with systemic diseases<br>Patients with medication that influences	Not stated

 © 2021. The Author(s).

Supplementary Table S2 (*Continued*)

Author(s)	Inclusion criteria	Exclusion criteria	Year
	Good oral health without caries or periodontal disease<br>Skeletal class I or mild classes II and III (ANB 0°–5°)	pain perception (analgesics, antidepressants, and/or anticonvulsants)<br>Severe malformations<br>Anatomy of the lingual side that would prevent lingual brackets being cemented in the lingual orthodontic group.	
Flores-Mir et al, 2018	All adult patients whose last orthodontic appointment before completion of treatment occurred between November 2014 and October 2016 were consecutively invited to participate in the survey during their debonding appointment		2014–2016
Christou et al, 2020	Males and females<br>Aged 12–30 years<br>Class I molar classification<br>Nonextraction treatment<br>Minor crowding (1–4 mm) in each dental arch<br>Treated in both dental arches<br>Pretreatment and posttreatment records available, including intraoral and extraoral composite photographs and lateral cephalometric radiographs taken within 6 months of initiation and completion of the treatment	Craniofacial discrepancies or syndromes<br>Previous orthognathic or cosmetic treatment<br>Previous esthetic treatment of lips<br>Previous traumas involving oral soft tissue<br>Patients with incomplete or poor quality of records<br>Unnatural posed smiles (i.e., a smile that is not reproducible, and therefore cannot be used as a reference for further measurements)	2015–2018
White, 2015	Class I molar and canine relationships<br>Nonextraction treatments<br>Mandibular crowding of 4 mm or less<br>No missing teeth (from second to second molar)	Anterior or posterior cross-bites<br>Anterior or lateral open bites<br>Maxillary overjet exceeding 3 mm<br>Impacted teeth	Not stated
Carrol, 2007	Upper and lower dental arches must be treated<br>The fixed appliances group must be treated with wires and brackets only (no quad helix, rapid palatal expander, etc.)<br>Must be 12 to 15 months into treatment<br>Must be 18 years of age or older<br>Must be willing to sign and give written informed consent in accordance with institutional and federal guidelines<br>Must be in good general health<br>Premolar and incisor extraction cases were acceptable	Not stated	Not stated
Nicholson, 2011	Comprehensive orthodontic treatment<br>At least 18 years old when treatment commenced<br>Treatment completed within past 2 years	Hybrid treatment involving both Invisalign and fixed appliances during most recent phase of orthodontics<br>Major health ailments that significantly affected activities of daily living (ADLs)	Not stated
Rucker, 2012	Not stated	Not stated	Not stated
Lawton, 2003	At least 18 years of age<br>Willing to sign informed consent<br>In good health<br>Able to be treated without extractions excluding a single lower incisor or third molars	Not stated	Not stated
Shalish et al, 2012	Age range 18–60 years<br>The need for comprehensive orthodontic treatment	Not stated	Not stated

 © 2021. The Author(s).
